# Cardiovascular, endothelial function, and immune markers in response to treatment with a polysaccharide in HIV^+^ adults in a randomized, double-blind placebo-controlled trial

**Published:** 2020-04-13

**Authors:** John E. Lewis, Steven E. Atlas, Muhammad H. Abbas, Ammar Rasul, Ashar Farooqi, Laura A. Lantigua, Frederick Michaud, Sharon Goldberg, Lucas C. Lages, Jinrun Gao, Oscar L. Higuera, Andrea Fiallo, Philip D. Harvey, Eduard Tiozzo, Judi M. Woolger, Stephanie Ciraula, Armando Mendez, Allan Rodriguez, Janet Konefal

**Affiliations:** ^1^Department of Psychiatry and Behavioral Sciences, University of Miami Miller School of Medicine, Miami, FL, USA; ^2^Department of Medicine, University of New Mexico School of Medicine, Albuquerque, NM, USA; ^3^Barclay’s, Inc., Wilmington, DE, USA; ^4^Department of Medicine, University of Miami Miller School of Medicine, Miami, FL, USA; ^5^Department of Family Medicine and Community Health, University of Miami Miller School of Medicine, Miami, FL, USA

**Keywords:** CD4^+^, endothelial function, HIV, hypertension, nutritional supplement, polysaccharide, rice bran arabinoxylan compound, systolic blood pressure

## Abstract

**Background and Aim:**

Given the ongoing problems of hypertension and endothelial dysfunction in the HIV population, the primary objective of the study was to assess the cardiovascular, endothelial function, and immune markers in response to rice bran arabinoxylan compound (RBAC) treatment in a sample of HIV^+^ adults on antiretroviral therapy (ART).

**Study Design:**

A randomized, double-blind placebo-controlled trial of 6 months was used to execute the study.

**Materials and Methods:**

Forty-seven subjects were enrolled and randomly assigned to one of two study conditions (*n*=22 RBAC and *n*=25 placebo) for 6 months with assessments at baseline and 3 and 6 months. A multivariate repeated measures analysis of variance model was used to assess the differences between RBAC and placebo groups in cardiovascular (systolic blood pressure), endothelial function (skin blood flow in response to nitric oxide), and immune (CD4^+^ cell count) markers from baseline to 6 months.

**Results:**

The effect of treatment (RBAC versus placebo) was significant (Wilks’ λ=0.92, F[3, 102]=3.07, *P*=0.03). The effect of time was significant (Wilks’ λ=0.10, F[2, 103]=474.6, *P*<0.001). The overall interaction between treatment and time was significant (Wilks’ λ=0.92, F[2, 103]=4.58, *P*=0.01). Time contrasts showed that a difference in the overall dependent variable did not occur from baseline to 3 months (F[1, 104]=2.7, *P*=0.10), marginally occurred from baseline to 6 months (F[1, 104]=3.2, *P*=0.08), and was significant from 3 to 6 months (F[1, 104]=6.43, *P*=0.01).

**Conclusions:**

The overall significant interaction suggests varying responses in the dependent variables between RBAC and placebo over time, which is being driven by systolic blood pressure, as it decreased in the RBAC group, but increased in the placebo group. In addition, CD4^+^ manifested a non-significant increase from baseline to 3 months then decreased from 3 to 6 months in the RBAC group, whereas it decreased at 3 months followed by a slight increase at 6 months in the placebo group. Skin blood flow in response to nitric oxide improved non-significantly overall in both groups, but worsened from 3 to 6 months in the placebo group. Thus, RBAC treatment may contribute to modest short-term improvements in systolic blood pressure, endothelial function, and CD4^+^ cell count, which could help improve the overall health profile of HIV^+^ adults.

**Relevance for Patients:**

Persons with HIV on ART suffer disproportionately from hypertension and endothelial dysfunction compared to the non-infected population, and conventional medical therapy does not alleviate these issues. RBAC is a safe, low-risk alternative that may help to improve the overall quality of life of these patients through modest improvements in these biomarkers plus CD4^+^ cell count.

## 1. Introduction

Over 1 million people are currently living with HIV (PLWH) in the United States. Once a lethal disease that was the leading cause of death among Americans aged 25-44 in the 1990s, HIV has now been transformed into a manageable chronic condition with advances in pharmacology, but it still remains among the ten leading causes of death for this age group [1,2]. Since the widespread initiation of antiretroviral therapy (ART), particularly the use of protease inhibitors, the HIV population finds itself with a higher rate of hypertension, the primary global risk factor for mortality, compared to HIV-uninfected individuals [3-5]. PLWH with hypertension also have a greater risk of cardiovascular disease (CVD), the leading worldwide cause of death, and all-cause mortality compared to non-infected adults with hypertension or normotensive PLWH [6-13]. Despite many recent medical advances in pharmacology and cardiology, clinical trials are still needed to counteract the rates of hypertension and related CVD in PLWH [14].

In addition to an elevated rate of hypertension, PLWH on ART also suffer from chronic microvascular disease and endothelial dysfunction, leading to increased inflammation [14]. In the non-HIV-infected population, the endothelium actively participates in both the innate and adaptive immune responses, including triggering pro- and anti-inflammatory responses and serving as antigen-presenting cells, but in PLWH these responses are not characterized [15,16]. Endothelial dysfunction is recognized as a starting point for HIV-related atherosclerosis, which ultimately leads to CVD [17]. Point of care screening for endothelial dysfunction is currently available; however, it has not yet become a part of routine HIV management. Given the elevated CVD risk among PLWH on ART, adding a validated endothelial dysfunction screening test to the standard of care in HIV disease could save lives through early intervention to prevent myocardial infarction and stroke.

Therapeutic nutritional supplementation holds promise as an adjunct to ART in the treatment of HIV disease. A wide range of beneficial physiologic effects is described in the literature, outlining clear biologic plausibility of nutritional therapies in PLWH. For example, antioxidants enhance mitochondrial energy production, decrease the release of lactic acid into the bloodstream, and enhance T and B lymphocyte proliferation [18], counteracting elevated reactive oxygen species, commonly known as oxidative stress [19]. In addition, several *in vitro* and *ex vivo* studies have shown rice bran arabinoxylan compound (RBAC) to possess a biologic response modifier effect on immune system function. RBAC has been found to enhance macrophage phagocytic activity and nitric oxide release and scavenge free radicals in a dose-dependent manner; thus, functioning as an antioxidant [20,21]. Our team found that RBAC demonstrated true immunomodulation by enhancing NK cell cytotoxicity, significantly changed nine out of 12 cytokines and growth factors, and was safe and tolerable among a sample of healthy adults [22]. We have also showed several clinically and statistically significant improvements, for example, alkaline phosphatase, platelets, neutrophils, neutrophil-lymphocyte ratio, and γ-glutamyl transferase, in response to 90 days of RBAC compared to placebo in adults with non-alcoholic fatty liver disease [23]. Recently, we demonstrated that RBAC produced clinically and statistically significant improvements in CD8^+^ cells and the CD4^+^/CD8^+^ ratio compared to placebo in a sample of HIV^+^ adults [24]. Although CD4^+^ count did not significantly increase, RBAC may have helped to sustain its level. In addition, CD4^+^ cells suffer attack by HIV, and their level can be influenced by non-disease factors such as host age, so values can be decidedly inconsistent [25,26]. Nonetheless, CD4^+^ cells are crucial to multiple functions in an optimal immune system and immunological memory [27], and routine CD4^+^ cell count is the standard of care assessment for HIV^+^ patients to determine infection progression and response to ART [25,28], despite expected inter-individual differences. Moreover, lower nadir CD4^+^ cell counts are related to a greater incidence of hypertension following the introduction of ART [4,29,30].

To the best of our knowledge, no clinical trial has been conducted to determine the effects of treatment with a polysaccharide nutritional supplement such as RBAC on cardiovascular and endothelial function markers in PLWH. Therefore, the purpose of this study is to determine the effect of 6 months of oral RBAC supplementation on these markers plus immune functioning as a validation marker from our previous analysis.

## 2. Materials and Methods

### 2.1. Subjects

This clinical trial was approved by the University of Miami Institutional Review Board for human subjects research (registry number: NCT02214173; available at: https://www.clinicaltrials.gov/ct2/show/NCT02214173). Possible participants were recruited in January 2015-October 2015 from physician referrals, the Medical Wellness Center, and the Departments of Psychiatry and Behavioral Sciences and Medicine at the University of Miami Miller School of Medicine, where the study was conducted. Inclusion criteria were (a) 18 years of age and above; (b) diagnosed with HIV; (c) CD4^+^ T cell count nadir 50-250 µL; (d) taking a stable ART regimen ≥6 months before and during study participation; (e) intention to stay on current medication regimen while enrolled in the study; (f) not taking a lipid-lowering medication for at least 3 months before starting the study; (g) the previous use of comparable polysaccharide dietary supplements allowed, but stopped 2 weeks before and for the remainder of the study; (h) inclination to follow all study procedures and assessments; and (i) capable of giving informed consent. Exclusion criteria were (a) currently participating in a different study investigating a comparable dietary supplement; (b) established allergy or sensitivity to rice, rice bran, mushrooms, or related food items; (c) any gastrointestinal ailments that could prevent uncertain absorption of the study supplement; (d) other medical issues that might make study participation difficult, for example, recent heart attack or stroke or chronic kidney disease; (e) existing immunomodulatory drug use, for example, interferon; (f) active chemotherapy; (g) multiple drug resistance to ART; (h) currently smoking; (i) serious anemia or other medical complication that would interfere with a safe blood draw; (j) bleeding disorder; or (k) currently pregnant or trying to conceive.

Possible subjects (*n*=73) were screened for study criteria. Qualified subjects (*n*=47) who met criteria were enrolled in the study after signing informed consent and HIPAA forms. Subjects were then enrolled to either (a) RBAC (*n*=22) or (b) placebo (*n*=25) study condition based on a random permutation table. Research staff and participants were blind to who received which treatment till after the data were analyzed. Daiwa Health Development (Gardena, CA, USA) provided the RBAC and placebo labeled as Protocol A and Protocol B, and one employee of Daiwa was responsible for maintaining the blind of the treatment assignment. Once participants were randomized, they were provided an assessment schedule for each study visit (i.e., baseline and 3 and 6 months), including the appointment for the blood draw for CD4^+^. Participants received $40 for the completion of each study visit, including all assessments. Thirty-four participants completed the study after ten withdrew at 3 months and three more withdrew at 6 months.

### 2.2. Intervention

All participants were directed to take two capsules 3 times/day (3 g/day total) for the entire 6-month trial period, irrespective of group assignment. Each RBAC capsule contained 500 mg of the active ingredient. Placebo capsules were not recognizable from the RBAC capsules and contained cellulose. Participants were not given any advice about diet, exercise, or medications. Consistent with the inclusion criterion, participants were told not to take any similar type of dietary supplement with mushroom ingredients or known immunomodulatory effects for the 2 weeks before the baseline assessment and throughout the 6-month trial period. From a side effect perspective, RBAC is similar to eating any other form of rice bran, and we have not documented any adverse events of RBAC in our previous studies [22-24]. Daiwa states that RBAC is a water-soluble rice bran extract that has been hydrolyzed by shiitake mushroom enzymes, and the final product for sale contains microcrystalline cellulose, hypromellose, sucrose fatty acid ester, gellan gum, and potassium acetate.

### 2.3. Outcomes and assessments

At baseline, participants completed sociodemographics and health/medical history questionnaires. Participants recorded their current medications and were asked to document any changes in type or amount during the trial period. Assessments were administered at baseline and at the end of 3 and 6 months (±1 week). Assessments were selected based on: (a) appropriateness for the patient population; (b) simplicity of use and interpretation; (c) the previous usage by study staff; and (d) inclusion of multiple techniques (i.e., self-report and biological values) to enhance the overall validity of the study.

### 2.4. Immune function

CD4^+^ cell count (cells/uL) was obtained using flow cytometry with BD FACSCount™ at each assessment.

### 2.5. Cardiovascular and endothelial function

The ANS-1 (LD Technology, Miami, FL, USA) is an FDA-approved and patented system that contains three assessment modules: (a) a sympathetic skin response (SSR) device, (b) a plethysmography sensor, and (c) a blood pressure device, which provides assessments of cardiac autonomic nervous system function, vascular dynamics, heart rate variability, and an overall cardiometabolic risk score. The ANS-1 SSR module assesses sudomotor function by generating a low-voltage signal with a constant weak direct current that is fed to the active electrode. The current ultimately stimulates the nicotinic muscarinic receptors (M-receptors) in the skin and sweat glands. In endothelial cells, M-activation increases nitric oxide that causes relaxation and vasodilation in adjacent vascular smooth muscle. In the sweat gland, the activation of M-receptors depolarizes the cell’s membrane to drive sweat production. The change in sweat production and blood flow affects the electrical conductance of the skin, which is measured by the ANS-1 as sudomotor function. The plethysmograph provides information about arterial stiffness and endothelial function. The data from the pulse oximeter and oscillatory blood pressure monitor are used to assess cardiac performance, including heart rate and systolic and diastolic blood pressure. For a complete description of the technical specifications and algorithmic operations of the ANS-1 system, please refer to the details in our most recent paper [31].

In two separate studies in persons with type 2 diabetes and retinopathy, we showed that ANS-1 markers were highly correlated with glucose, insulin, and C-reactive protein, among others, and the system could discriminate between type 2 diabetes and retinopathy patients and healthy participants [31,32]. We also demonstrated the system’s accuracy for the assessment of body composition and cardiac output in additional studies [33]. Other investigators have found the ANS-1 to be accurate in detecting peripheral distal neuropathy symptoms [34] and insulin resistance [35]. The current study is the first attempt to use the ANS-1 system in PLWH to evaluate blood pressure and endothelial function to determine their response to RBAC treatment. Systolic blood pressure (the cardiovascular function marker) and skin blood flow in response to nitric oxide (the endothelial function marker) were selected as two of the three dependent variables (CD4^+^ being the third variable) for analysis in the statistical model.

As per standard protocol, every team member was trained by the manufacturer. Participants were instructed to sit in a chair in front of the ANS-1 unit, bare feet on the metal plates, right index finger in the pulse oximeter, and blood pressure cuff on the left arm. After entering demographic, anthropometric, and physical activity information, the assessment was initiated for 2 min while the participant was sitting down. Then, the participant was asked to perform the Valsalva maneuver by squeezing the nose with the left hand while trying to breathe out for 15 s with the mouth closed to build pressure. After releasing the nose, the participant was told to breathe deeply for 30 s, inhaling and exhaling for 5 s each. Finally, the participant was required to stand up until the assessment was completed, while straightening the left arm to the side and keeping the index finger in the pulse oximeter. The entire ANS-1 assessment lasted about 12 min.

### 2.6. Descriptive and control variables

Sociodemographics such as age, sex, race/ethnicity, education, and marital status were recorded at baseline. The medical and health assessment included an emphasis on opportunistic infections, respiratory diseases, diabetes, CVD, oral diseases, history of cancer, and drug, alcohol, and tobacco use. History of ART use was recorded and confirmed with the medical record. Participants were queried about the incidence of opportunistic infections and hospitalizations at the follow-up visits. ART-related effects and non-HIV related medications were recorded.

### 2.7. Adverse events

Potential adverse effects were discussed with each participant during the informed consent process. Participants were actively observed for adverse events throughout the intervention period.

### 2.8. Statistical analysis

Frequency and descriptive statistics were calculated on all variables. We used the multivariate repeated measures analysis of variance model to assess changes in the dependent variables of cardiovascular (systolic blood pressure), endothelial function (skin blood flow in response to nitric oxide), and immune (CD4^+^ cell count) markers from baseline to 6 months between the RBAC and the placebo groups. Systolic blood pressure, skin blood flow in response to nitric oxide, and CD4^+^ cell count were the dependent variables chosen for the model based on their statistical and clinical significance from our own work and of that in the extant literature. Many other variables were collected that could have been selected for this analysis, but only these three variables were used in the subsequent modeling for expediency and relevance. Multivariate tests (Wilks’ λ) were computed for the between-subjects (treatment group; RBAC vs. placebo) and within-subjects (time; baseline vs. 3 months versus 6 months) factors and the interaction between treatment group and time. The primary test of interest was the interaction between treatment group and time to determine if the treatment had differential effects for RBAC and placebo groups over time. Contrasts (analysis of variance) were also calculated to simultaneously compare the dependent variables at 1 time point to all subsequent time points to determine where differences may have occurred. Assuming significant contrasts, univariate paired-sample *t*-tests were used to determine changes between any 2 time points for both RBAC and placebo groups. Data were analyzed using SAS 9.3 (SAS, Durham, NC, USA), and α<0.05 was considered statistically significant.

## 3. Results

### 3.1. Sociodemographics

Table 1 shows the descriptive sociodemographic information of the sample, including age, sex, race/ethnicity, education, and marital status. No significant differences were detected between the RBAC and placebo groups. All descriptive data for comorbid disorders, medication use, liver enzymes, kidney function, and safety can be reviewed in our previously published paper [24], and we did not include that information in this paper for brevity.

**Table 1:** Sociodemographic characteristics of the sample.

Variable	Category	RBAC (*n*=22)	Placebo (*n*=25)	Statistic
Age	-	M=50.3, SD=10.5, R=18, 64	M=48.1, SD=9.8, R=24, 66	t=0.8 (45), p=0.45
Sex	Male	9 (41%)	13 (52%)	Χ^2^=0.6 (1), p=0.45
	Female	13 (59%)	12 (48%)	
Race/Ethnicity	White, non-hispanic	-	2 (8%)	χ^2^=0.6 (1), p=0.45
	Black, non-hispanic	16 (73%)	16 (64%)	
	Hispanic	6 (27%)	6 (24%)	
	Other	-	1 (4%)	
Education	Up to high school	7 (32%)	5 (21%)	χ^2^=4.4 (3), p=0.22
	High school graduate	7 (32%)	6 (25%)	
	Post high school/Some college	8 (36%)z	9 (38%)	
	College graduate	-	4 (17%)	
Marital Status	Never married	12 (55%)	18 (72%)	χ^2^=4.0 (2), p=0.14
	Married	1 (5%)	3 (12%)	
	Widowed/divorced	9 (41%)	4 (16%)	

M: Mean, SD: Standard deviation, R: Range, RBAC: Rice bran arabinoxylan compound

### 3.2. Cardiovascular, endothelial function, and immune markers

Table 2 shows the descriptive data for systolic blood pressure (mm Hg), skin blood flow in response to nitric oxide (≥900 mV), and CD4^+^ cell count (cells/uL) for both groups at baseline and 3 and 6 months. The group effect of RBAC versus placebo was significant (Wilks’ λ=0.92, F[3, 102]=3.07, *P*=0.03). The effect of time was also significant (Wilks’ λ=0.10, F[2, 103]=474.6, *P*<0.001). The overall interaction between RBAC versus placebo and time was also significant (Wilks’ λ=0.92, F[2, 103]=4.58, *P*=0.01), suggesting that the changes in the dependent variables over the course of the intervention were different between the two groups. Time contrasts showed that no treatment-related difference was found from baseline to 3 months (F[1, 104]=2.7, *P*=0.10), was marginally significant from baseline to 6 months (F[1, 104]=3.2, *P*=0.08), and was significant from 3 to 6 months (F[1, 104] = 6.43, *P*=0.01), suggesting no effect of the treatment initially (baseline to 3 months), but a significant effect of the treatment toward the end of the intervention (3-6 months). Univariate *t*-tests for systolic blood pressure, skin blood flow in response to nitric oxide, and CD4^+^ cell count showed no statistically significant changes between any 2 time points for both groups, other than an increase in skin blood flow in response to nitric oxide in the placebo group from 3 to 6 months (t[14]=2.5, *P*=0.03), which corresponds to a worsening of the response.

**Table 2:** Biomarkers at baseline and 3 and 6 months.

Measure	Time	RBAC (*n*=22)	Placebo (*n*=25)
Systolic blood pressure (mm Hg)	Baseline	130.9±17.6 (103, 171)	133.5±15.7 (96, 166)
	3 months	128.2±13.8 (109, 157)	133±16.6 (109, 174)
	6 months	127.7±12.9 (111, 159)	138.8±18.5 (122, 190)
Skin blood flow in response to nitric oxide (≥900 mV)	Baseline	67.7±20.1 (63, 98)	65.5±16.5 (29, 97)
	3 months	62.9±20.6 (25, 95)	51.1±16.5 (20, 95)
	6 months	60.9±21.9 (20, 94)	55.6±18.9 (13, 83)
CD4^+^ cell count (cells/uL)	Baseline	705.86±372.33 (107, 1559)	621.36±366.73 (72, 1341)
	3 months	800.31±708.74 (110, 3136)	548.52±381.23 (9, 1335)
	6 months	704.18±358.68 (187, 1329)	605.47±435.65 (1, 1330)

Values are mean±standard deviation (minimum, maximum). For systolic blood pressure and skin blood flow in response to nitric oxide, a decrease in value from 1 time point to another would typically be clinically beneficial, whereas for CD4^+^ cell count an increase in value from 1 time point to another would typically be clinically beneficial. RBAC: Rice bran arabinoxylan compound

## 4. Discussion

In the current study, we found an overall significant interaction effect, suggesting simultaneous changes in our dependent variables in the RBAC group compared to the placebo group. Although not statistically significant, the changes in systolic blood pressure appeared to at least partially be driving the overall significant interaction effect. Systolic blood pressure decreased in the RBAC group, whereas systolic blood pressure increased in the placebo group. Skin blood flow in response to nitric oxide decreased overall from baseline to 6 months in both groups, but in the placebo group it significantly increased (worsened) from 3 to 6 months. In addition, the CD4^+^ count increased from baseline to 3 months and then returned to baseline from 3 to 6 months in the RBAC group, whereas the placebo group decreased at 3 months and then slightly increased at 6 months, further contributing to the significant interaction effect.

Nonetheless, additional study is required to determine if RBAC treatment may more conclusively contribute to short-term improvements in systolic blood pressure and skin blood flow in response to nitric oxide (baseline to 6 months) and CD4^+^ cell count (baseline to 3 months), which could contribute to an overall improved health profile of HIV^+^ adults, a population at increased risk of CVD. Our finding is modestly important, given that studies indicate that PLWH taking ART have a higher rate of hypertension and suffer from endothelial dysfunction and chronic inflammation, placing them at increased risk of CVD and all-cause mortality compared to the non-HIV-infected population [5,7-9,14]. In addition, PLWH are a model of accelerated aging due to immunosenescence [24], which has also been theorized as the reason for their heightened risk of CVD [36]. Moreover, our results are at least partially consistent with the previous well-established relationships among atherosclerosis, increased CVD risk, endothelial dysfunction, and immune dysregulation in PLWH [8,37-39].

The modest improvements in our dependent variables in the RBAC group are important and may be supportive of other work that has shown how the cardiovascular, endothelial, and immune systems are dependent on each other [15]. Of note, this is a small study and some of the changes that were not statistically significant in the RBAC group would likely be significant with larger sample sizes, consistent with those that would typically be used in a Phase III trial. The ability of RBAC to have a positive impact on these biomarkers in a relatively short period of time could be significant for counteracting disease progress in the HIV population, particularly since patients now live with HIV for many years and counteracting their heightened immunosenescence is clinically important. Another recent study showed little relationship between baseline CD4^+^ cell count or change in CD4^+^ cell count from baseline to 4 months and autonomic function after initiation of ART, with the exception of changes in high and low frequency responses during the head-up tilt test [40].

To the best of our knowledge, the current study is the first one to simultaneously assess cardiovascular, endothelial function, and immune markers in response to a 6-month trial of a nutritional supplement of any kind, particularly this type of polysaccharide as RBAC. Other studies in PLWH taking ART have shown significant responses to certain nutrients or phytonutrients on outcome variables similar to the current investigation. For example, daily ingestion of 5 mg of folinic acid for 4 weeks was superior to placebo in improving vascular reactivity, a marker of endothelial function [41]. A second similar recent clinical trial of subjects undergoing aerobic exercise plus daily consumption of 5 mg of folinic acid or placebo showed that endothelial function according to venous occlusion plethysmography was better in the folinic acid group [42]. Another short-term trial in PLWH who consumed either dark chocolate or a chocolate placebo showed significant improvements in large artery elasticity in the dark chocolate group [43]. A large trial of HIV-positive women showed that those taking a multivitamin dietary supplement were 38% less likely to develop hypertension (systolic blood pressure ≥140 mm Hg or diastolic blood pressure ≥90 mm Hg) during pregnancy than those on placebo [44]. Finally, another clinical trial showed that a micronutrient supplement significantly increased the absolute CD4 count (24%) compared to placebo (0%) [45]. Thus, the findings of the current study are consistent with other studies demonstrating that certain nutrients or dietary supplements can have beneficial effects on various markers of endothelial function, blood pressure, and CD4 count, which can benefit the HIV patient population.

### 4.1. Limitations

Nutritional supplement clinical trials are typically difficult to demonstrate efficacy in PLWH because of the occurrence of multiple medical comorbidities. Thus, confounding from these comorbidities may be an uncontrolled factor in the study’s results. In addition to comorbidities, other behaviors, such as diet, exercise, stress management, and sleep, may have at least partially influenced the results of our study. While we did not utilize a compliance measure, the study team made weekly check-in calls with participants to discuss any problems and to encourage adherence to the protocol. Thus, our participants were consistently and frequently reminded about the importance of their adherence to the study’s procedures. The typical PLWH need frequent medical observation with various clinician specialties, so time and interest for taking part in research may also be a factor that hinders overall scientific progress. In addition, PLWH have a usual high pill burden with typical side effects, which may also discourage this population from participating in clinical trials. Nutritional supplementation is distinct from routine HIV care, making it difficult to recruit interested, willing, and reliable study candidates. However, even with all these recruiting obstacles, the effects of RBAC in this study are modestly favorable. The small number of subjects and minor attrition rate could have negatively affected the study’s power, which could have also limited the current findings. Finally, RBAC led to a slight improvement in immune functioning in CD4^+^ at 3 months, but it was not sustained at 6 months. Thus, future research should consider whether a higher RBAC dose or an ascending titration schedule would lead to more sustained benefits. Furthermore, a longer intervention period, for example, 12 months, would better evaluate how RBAC may help to improve the overall health profile of a population at increased risk of hypertension, endothelial dysfunction, and CVD. While these very modest short-term improvements are beneficial, PLWH need long-term solutions to counteract their greater risks of chronic diseases.

## 5. Conclusions

A nutritional supplement that could attenuate hypertension and endothelial dysfunction in PLWH on ART would be beneficial, but studies are limited. HIV is now considered a chronic disease; hence, an efficacious nutritional supplement could be an important adjunct to conventional HIV treatment. Based on the overall significant interaction in the changes in the dependent variables in the current study, RBAC may be such a supplement. Given that RBAC is an all-natural product with antioxidant and immunomodulatory properties, without any known adverse effects or interactions with pharmaceuticals, it should be further studied in PLWH.

In addition, the use of the ANS-1 system, a noninvasive, validated method of evaluating endothelial function, in PLWH taking ART should be further studied as part of a CVD risk reduction strategy. The current results support our previous work with the ANS-1 and its usefulness in the primary care setting [33].

## References

[1] Centers for Disease Control and Prevention. HIV Prevalence Estimates--United States 2006 (2008). MMWR Morb Mortal Wkly Rep.

[2] Centers for Disease Control and Prevention (2015). HIV Surveillance Report.

[3] Gazzaruso C, Bruno R, Garzaniti A, Giordanetti S, Fratino P, Sacchi P (2003). Hypertension among HIV Patients:Prevalence and Relationships to Insulin Resistance and Metabolic Syndrome. J Hypertens.

[4] Peck RN, Shedafa R, Kalluvya S, Downs JA, Todd J, Suthanthiran M (2014). Hypertension, Kidney Disease, HIV and Antiretroviral Therapy among Tanzanian Adults:A Cross-sectional Study. BMC Med.

[5] Xu Y, Chen X, Wang K (2017). Global Prevalence of Hypertension among People Living with HIV:A Systematic Review and Meta-analysis. J Am Soc Hypertens.

[6] Sinha A, Ma Y, Scherzer R, Hur S, Li D, Ganz P (2016). Role of t-cell Dysfunction, Inflammation, and Coagulation in Microvascular Disease in HIV. J Am Heart Assoc.

[7] Iantorno M, Schar M, Soleimanifard S, Brown TT, Moore R, Barditch-Crovo P (2017). Coronary Artery Endothelial Dysfunction is Present in HIV-Positive Individuals without Significant Coronary Artery Disease. AIDS.

[8] Hove-Skovsgaard M, Gaardbo JC, Kolte L, Winding K, Seljeflot I, Svardal A (2017). HIV-Infected Persons with Type 2 Diabetes Show Evidence of Endothelial Dysfunction and Increased Inflammation. BMC Infect Dis.

[9] Nuesch R, Wang Q, Elzi L, Bernasconi E, Weber R, Cavassini M (2013). Risk of Cardiovascular Events and Blood Pressure Control in Hypertensive HIV-Infected Patients:Swiss HIV Cohort Study (SHCS). J Acquir Immune Defic Syndr.

[10] Triant VA, Lee H, Hadigan C, Grinspoon SK (2007). Increased Acute Myocardial Infarction Rates and Cardiovascular Risk Factors among Patients with Human Immunodeficiency Virus Disease. J Clin Endocrinol Metab.

[11] Phillips AN, Carr A, Neuhaus J, Visnegarwala F, Prineas R, Burman WJ (2008). Interruption of Antiretroviral Therapy and Risk of Cardiovascular Disease in Persons with HIV-1 Infection:Exploratory Analyses from the Smart Trial. Antivir Ther.

[12] Armah KA, Chang CC, Baker JV, Ramachandran VS, Budoff MJ, Crane HM (2014). Prehypertension, Hypertension, and the Risk of Acute Myocardial Infarction in HIV-Infected and-Uninfected Veterans. Clin Infect Dis.

[13] Bloomfield GS, Hogan JW, Keter A, Holland TL, Sang E, Kimaiyo S (2014). Blood Pressure Level Impacts Risk of Death Among HIV Seropositive Adults in Kenya:A Retrospective Analysis of Electronic Health Records. BMC Infect Dis.

[14] Fahme SA, Bloomfield GS, Peck R (2018). Hypertension in HIV-Infected Adults:Novel Pathophysiologic Mechanisms. Hypertension.

[15] Mai J, Virtue A, Shen J, Wang H, Yang XF (2013). An Evolving New Paradigm:Endothelial Cells--Conditional Innate Immune Cells. J Hematol Oncol.

[16] Choi J, Enis DR, Koh KP, Shiao SL, Pober JS (2004). T Lymphocyte-endothelial Cell Interactions. Annu Rev Immunol.

[17] Auclair M, Afonso P, Capel E, Caron-Debarle M, Capeau J (2014). Impact of Darunavir, Atazanavir and Lopinavir Boosted with Ritonavir on Cultured Human Endothelial Cells:Beneficial Effect of Pravastatin. Antivir Ther.

[18] Kalebic T, Kinter A, Poli G, Anderson ME, Meister A, Fauci AS (1991). Suppression of Human Immunodeficiency Virus Expression in Chronically Infected Monocytic Cells by Glutathione, Glutathione Ester, and n-acetylcysteine. Proc Natl Acad Sci U S A.

[19] Ivanov AV, Valuev-Elliston VT, Ivanova ON, Kochetkov SN, Starodubova ES, Bartosch B (2016). Oxidative Stress during HIV Infection:Mechanisms and Consequences. Oxid Med Cell Longev.

[20] Ghoneum M, Matsuura M (2004). Augmentation of Macrophage Phagocytosis by Modified Arabinoxylan Rice Bran (mgn-3/biobran). Int J Immunopathol Pharmacol.

[21] Tazawa K, Namikawa H, Oida N, Masada M, Maeda H (2000). Scavenging Activity of Modified Arabinoxylane from Rice Bran (biobran/mgn-3) with Natural Killer Cell Activity on Free Radicals. Biotherapy.

[22] Ali K, Melillo A, Leonard S, Asthana D, Woolger J, Wolfson A (2012). An Open-label, Randomized Clinical Trial to Assess the Immunomodulatory Activity of a Novel Oligosaccharide Compound in Healthy Adults. Funct Foods Health Dis.

[23] Lewis JE, Atlas SE, Higuera OL, Fiallo A, Rasul A, Farooqi A (2018). The Effect of a Hydrolyzed Polysaccharide Dietary Supplement on Biomarkers in adults with Nonalcoholic Fatty Liver Disease. Evid Based Complement Alternat Med.

[24] Lewis JE, Atlas SE, Abbas MH, Rasul A, Farooqi A, Lantigua LA (2018). The Novel Effects of a Hydrolyzed Polysaccharide Dietary Supplement on Immune, Hepatic, and Renal Function in Adults with HIV in a Randomized, Double-blind Placebo-control Trial. J Diet Suppl.

[25] Nanzigu S, Kiguba R, Kabanda J, Mukonzo JK, Waako P, Kityo C (2013). Poor Immunological Recovery among Severely Immunosuppressed Antiretroviral Therapy-Naive Ugandans. HIV AIDS (Auckl).

[26] Merci NM, Emerence U, Augustin N, Habtu M, Julie I, Angelique T (2017). CD4+Cells Recovery in HIV Positive Patients with Severe Immunosuppression at Haart Initiation at Centre Medico-Social Cor-Unum, Kigali. Pan Afr Med J.

[27] Zhu J, Yamane H, Paul WE (2010). Differentiation of Effector CD4 t Cell Populations (*). Annu Rev Immunol.

[28] Twizerimana AP, Mwatha J, Musabyimana JP, Kayigi E, Jde DH, Karanja SM (2014). Immunological Profiles in HIV Positive Patients Following Haart Initiation in Kigali, Rwanda. East Afr Med J.

[29] Manner IW, Troseid M, Oektedalen O, Baekken M, Os I (2013). Low Nadir CD4 Cell Count Predicts Sustained Hypertension in HIV-Infected Individuals. J Clin Hypertens (Greenwich).

[30] Crane HM, Van Rompaey SE, Kitahata MM (2006). Antiretroviral Medications Associated with Elevated Blood Pressure among Patients Receiving Highly Active Antiretroviral Therapy. AIDS.

[31] Lewis JE, Atlas SE, Rasul A, Farooqi A, Lantigua L, Higuera OL (2017). New Method of Sudomotor Function Measurement to Detect Microvascular Disease and Sweat Gland Nerve or Unmyelinated c Fiber Dysfunction in Adults with Retinopathy. J Diabetes Metab Disord.

[32] Lewis JE, Lantigua L, Atlas SE, Lopez J, Mendez A, Goldberg S (2014). A Cross-sectional Assessment to Detect Type 2 Diabetes with Endothelial and Autonomic Nervous System Markers Using a Novel System. J Diabetes Metab Disord.

[33] Lewis JE, Tannenbaum SL, Gao J, Melillo AB, Long EG, Alonso Y (2011). Comparing the Accuracy of ES-BC, EIS GS, and ES Oxi on Body Composition, Autonomic Nervous System Activity, and Cardiac Output to Standardized Assessments. Med Devices (Auckl).

[34] Gandhi P, Rao G (2015). Detection of Neuropathy Using a Sudomotor Test in Type 2 Diabetes. Degener Neurol Neuromuscul Dis.

[35] De Souza AL, Batista GA, Alegre SM (2017). Assessment of Insulin Sensitivity by the Hyperinsulinemic Euglycemic Clamp:Comparison with the Spectral Analysis of Photoplethysmography. J Diabetes Complications.

[36] Maffongelli G, Alteri C, Gentilotti E, Bertoli A, Ricciardi A, Malagnino V (2016). Impact of HIV-1 Tropism on the Emergence of Non-aids Events in HIV-infected Patients Receiving Fully Suppressive Antiretroviral Therapy. AIDS.

[37] Freiberg MS, Chang CC, Kuller LH, Skanderson M, Lowy E, Kraemer KL (2013). HIV Infection and the Risk of Acute Myocardial Infarction. JAMA Intern Med.

[38] Hsue PY, Deeks SG, Hunt PW (2012). Immunologic Basis of Cardiovascular Disease in HIV-Infected Adults. J Infect Dis.

[39] Worm SW, De Wit S, Weber R, Sabin CA, Reiss P, El-Sadr W (2009). Diabetes Mellitus, Preexisting Coronary Heart Disease, and the Risk of Subsequent Coronary Heart Disease Events in Patients Infected with Human Immunodeficiency Virus:The Data Collection on Adverse Events of Anti-HIV Drugs (d:A:D study). Circulation.

[40] Chow D, Kocher M, Shikuma C, Parikh N, Grandinetti A, Nakamoto B (2012). Effects of Antiretroviral Therapy on Autonomic Function in Early HIV Infection:A Preliminary Report. Int J Med Sci.

[41] Grigoletti SS, Guindani G, Moraes RS, Ribeiro JP, Sprinz E (2013). Short-term Folinic Acid Supplementation Improves Vascular Reactivity in HIV-Infected Individuals:A Randomized Trial. Nutrition.

[42] Grigoletti SS, Ribeiro JP, Sprinz E, Ribeiro PA (2018). Short-Term Folinic Acid Supplementation and Aerobic Exercise Improve Vascular Reactivity in HIV-Infected Individuals. HIV Clin Trials.

[43] Teixeira A, Luzia LA, de Souza SJ, de Almeida Petrilli A, Pontilho PM, de Souza JM (2017). The Impact of Dark Chocolate Intake on Arterial Elasticity in Individuals with HIV/AIDS Undergoing Art:A Randomized, Double-blind, Crossover Trial. Food Funct.

[44] Merchant AT, Msamanga G, Villamor E, Saathoff E, O'Brien M, Hertzmark E (2005). Multivitamin Supplementation of HIV-positive Women during Pregnancy Reduces Hypertension. J Nutr.

[45] Kaiser JD, Campa AM, Ondercin JP, Leoung GS, Pless RF, Baum MK (2006). Micronutrient Supplementation Increases CD4 Count in HIV-infected Individuals on Highly Active Antiretroviral Therapy:A Prospective, Double-blinded, Placebo-controlled Trial. J Acquir Immune Defic Syndr.

